# Recent advances in gastrointestinal immunology

**Published:** 2016

**Authors:** Luke Materacki, David Al Dulaimi

**Affiliations:** *Department of Gastroenterology, Alexandra Hospital, Redditch, UK*

*Biancheri P et al* *.* ***Abnormal thymic stromal lymphopoietin expression in the duodenal mucosa of patients with coeliac disease.*** *Gut 2015;0:1-11*

The expression of epithelial-derived thymic stromal lymphopoietin (TSLP), a key cytokine modulator of gut immune tolerance, may be dysregulated in coeliac disease (CD). This study investigated TSLP expression along the clinical spectrum of CD and evaluated the *ex vivo *and *in vitro* effects of TSLP isoforms in CD mucosa. D2 biopsies from 64 patients with untreated CD, 50 patients with treated CD, 8 patients with refractory CD, 4 patients with potential CD and 43 control subjects were analysed. TSLP gene and protein expression was significantly lower in the epithelium and lamina propria of patients with untreated and refractory CD compared to the control, treated and potential CD groups (p<0.05). This occurred irrespective of the degree of mucosal damage and may be related to the expression of furin, an endopeptidase known to cleave TSLP, which was increased in the D2 mucosa of untreated CD compared with treated CD and controls (p<0.05). 

TSLP was associated with a number of immunological consequences. Long and short TSLP isomers downregulated the production of pro-inflammatory IFN-γ, IL-8 and T-bet, a Th1-specific transcription factor, when cultured with untreated CD biopsies *ex* *vivo* (p<0.05). Furthermore IL-8 was significantly decreased in the culture supernatants of treated CD (p<0.005) and controls (p<0.05) compared to untreated CD. 

Overall TSLP deficiency in active CD may cause upregulation of the Th1 mucosal immune response with subsequent mucosal damage. Restoring TSLP function may represent a novel therapeutic target in patients with refractory CD.

*Cheung K et al.* ***CD31 signals confer immune privilege to the vascular endothelium.*** *Proc Natl Acad Sci U S A 2015;112(43):E5815-24.*

Maintaining vascular integrity during inflammation is important however the molecular mechanisms protecting endothelial cells (ECs) from apoptosis are poorly understood. This study investigated the role of CD31 (platelet endothelial cell adhesion molecule-1) in protecting ECs from immunological stress in a murine model. ECs from CD31-deficient mice underwent a significantly higher rate of apoptosis compared to wild type ECs when exposed to TNFα. Similar findings were observed in a model of T-cell mediated cytotoxicity. Furthermore, antibody inhibition of CD31 activation increased the rate of TNFα-induced apoptosis of ECs. Overall, CD31 expression in ECs appears necessary to confer cytoprotection against TNFα-induced apoptosis and cytotoxic T-lymphocyte mediated cytolysis. Potential mechanisms for CD31-mediated EC survival were evaluated. Activation of the Erk/Akt pathway was CD31-dependent and may confer resistance from TNFα-induced apoptosis in ECs. CD31 promoted a prosurvival transcriptional cascade downstream of TNFα. 

Loss of CD31 expression by skin allografts led to increased rejection and extensive vascular damage compared with wild type skin grafts. Notably *In vivo,* CD31 gene transfer conferred resistance to immune-mediated rejection in CD31^-^ pancreatic β cells grafted in fully allergenic recipients. Overall CD31, expressed in high levels by ECs, appears to confer immune privilege during extrinsic apoptotic stimuli so that vascular integrity is maintained.

*Fitzmaurice K et al.* ***Additive effects of HLA alleles and innate immune genes determine viral outcome in HCV infection.*** *Gut 2015; 64(5):813-9.*

The host factors which influence outcomes following hepatitis C virus (HCV) infection are poorly understood. This cohort study investigated the effect of genetic variability in the innate and adaptive immune systems on HCV (genotype-1b) infection in 319 Irish females exposed following receipt of contaminated anti-D immunoglobulin in 1977. The presence of HLA-A*03, -B*15, -B*27, -C*01, -C*12, -DRB1*01:01, -DRB1*05:01 and the protective IFNL3 rs12979860 genotype was significantly associated with viral clearance (p<0.05). Interestingly, HLA-A*03 was only significantly associated with viral clearance in patients with the protective IFNL3 rs12979860 genotype and similarly HLA-B*27 and -C*01 required the deleterious IFNL3 genotype to demonstrate a significant effect on viral clearance. Conversely HLA-C*04, -C*09, -DQB1*02:01, -DRB1*03:01, KIR2DS3 and the rs12979860 IFNL3 'T' allele were associated with chronic HCV infection (p<0.05). 

An independent and additive effect of HLA class I and II alleles and IFNL3 in relation to viral infection was observed. These findings were corroborated by further analysis of an independent heterogeneous Swiss cohort of 416 patients with HIV and HCV coinfection. The authors conclude that interplay between the adaptive and innate immune response is important to achieve HCV clearance. 

*Lehouritis P et al.* ***Local bacteria affect the efficacy of chemotherapeutic drugs.*** *Sci Rep 2015;5:14554*

An understanding of the factors which influence the efficacy of chemotherapy is important to optimise therapeutic outcome. The effect of biotransformation of chemotherapeutics during cancer therapy is unclear. This *in vitro* study evaluated whether the presence of non-pathogenic *E. coli* or *Listeria welshimeri* influenced the efficacy of 30 chemotherapy agents in various murine cancer cell lines. *E.coli* increased the cytotoxicity of tegafur, fludarabine de phosphate, 5-fluorocytosine, 6-mercaptopurine-2'-deoxyribosine, AQ4N, CB1954 and decreased the cytotoxicity of cladribine, vibarabine, gemcitabine, doxorubicin, daunorubicin, idarubicin, etoposide phosphate, mitoxantrone, B-lapachone and menadione. *Listeria welshimeri* increased the cytotoxicity of fludarabine de phosphate and CB1954 and reduced the cytotoxicity of cladribine and daunorubicin*. *A dose response was identified using different bacterial concentrations (p<0.01). The observed microbial effects on chemotherapy were abolished with heat treatment suggesting an enzymatic mechanism (p<0.001). All drugs demonstrated new chromatogram peaks after bacterial exposure indicating biotransformation.

This study helps to further clarify microbial-drug interactions and highlights a role for manipulation of the microbiome to optimise chemotherapy outcomes.

*Reyes A et al.* ***Gut DNA viromes of Malawian twins discordant for severe acute malnutrition.*** *Proc Natl Acad Sci U S A 2015;112(38):11941-6.*

The human gut microbiome appears to have a key role in the pathogenesis of several systemic diseases. Childhood malnutrition may be associated with persistent immaturity of gut microbiota and poor health in later life. This time-series metagenomic study of DNA isolated from virus-like particles investigated the faecal DNA virome in 8 pairs of monozygotic and dizygotic Malawian twins concordant for healthy growth and 12 twin pairs discordant for severe acute malnutrition (kwashiorkor (n=6) or marasmus (n=6)) from birth to 30 months old. Each twin pair was treated with peanut-based ready-to-use therapeutic food (RUTF) for 2 to 8 weeks. 

The composition of the faecal DNA virome was dictated to a greater extent by age than genetics. Twins with severe acute malnutrition demonstrated less variation in their faecal DNA virome compared to healthy twins and this difference endured during and following treatment with RUTF. Viral contigs discriminating age, families and the presence of healthy concordant twins or twins discordant for severe acute malnutrition were identified. Transplantation of faecal microbiota from twins discordant for kwashiorkor into germ-free mice led to the transmission of discordant weight loss, metabolic phenotypes and an enteropathy characterised by small bowel and colonic epithelial disruption. Several viruses identified in the human donor faeces were not subsequently isolated in recipient gnotobiotic mice faeces and therefore the mechanism leading to recipient disease requires further investigation. Potential therapies aimed at accelerating the maturation of gut microbiota in patients with severe acute malnutrition should be investigated.

